# New approaches to tackle a rising problem: Large-scale methods to study antifungal resistance

**DOI:** 10.1371/journal.ppat.1012478

**Published:** 2024-09-05

**Authors:** Philippe C. Després, Rebecca S. Shapiro, Christina A. Cuomo

**Affiliations:** 1 Infectious Disease and Microbiome Program, Broad Institute of MIT and Harvard, Cambridge, Massachusetts, United States of America; 2 Department of Molecular Microbiology and Immunology, Brown University, Providence, Rhode Island, United States of America; 3 Department of Molecular and Cellular Biology, University of Guelph, Guelph, Ontario, Canada; University of Maryland, Baltimore, UNITED STATES OF AMERICA

## Introduction

Fungi are important pathogens responsible for millions of infections and deaths every year through disease and food insecurity [1,2]. Drug-resistant fungi are especially concerning due to the limited therapeutic arsenal available to fight them. Despite this, our ability to predict what genomic changes cause antifungal resistance and by which mechanisms remain limited. Here, we review recent advances in genomics and large-scale experimental assays to catalogue such mutations and study the biology associated with them. We discuss the progress in assembling databases of genes and variants implicated in resistance and virulence as well as the important role these might play in developing new diagnostics tools for use in the clinic and the field.

## Why the urgency?

The scarcity of antifungal compounds available to treat infections makes the rise in incidence of both intrinsic and acquired resistance in clinical and field isolates an alarming situation [3]. Key to facing this challenge is understanding how resistance to antifungals arises. In some species infecting humans, like *Aspergillus fumigatus*, resistance can be connected to the use of antifungals in agriculture [4], showing that the issue of resistance exists in a “One Health” context [2]. While some of the causal genes are known (e.g., ERG11/CYP51, PDR1, FKS genes, TUB genes), resistance can still arise through a variety of mechanisms: coding sequence or promoter mutations, copy number alterations, aneuploidies or even epigenetic modifications, none of which have been fully cataloged [5]. Furthermore, we have yet to uncover the precise mechanisms by which some species are intrinsically more resistant to certain antifungals, like *Candida auris* [6].

This incomplete knowledge has important implications regarding next-generation approaches to tackle fungal pathogens. The switch to molecular diagnostics tools to detect resistance markers could accelerate the use of optimal treatment regimens but requires a deep understanding of the genotype-to-phenotype link [7]. As new compounds are being discovered and progress through clinical trials [8], we also have the opportunity to map out evolutionary pathways to resistance and characterize tolerance before these compounds see widespread use, to maximize their efficacy. Finally, understanding the trade-offs in growth rate, stress resistance, or virulence associated with resistance to a particular antifungal could also help uncover vulnerabilities unique to resistant strains, leading to new strategies to bypass resistance [9]. Rapid advances in sequencing, molecular biology, and bioinformatics provide opportunities to upscale or parallelize many experiments, which can improve efficiency and generate new knowledge on these important questions.

## Genome sequencing identifies causal resistance genes and retraces the evolution of pathogenic traits

**Fig 1:**
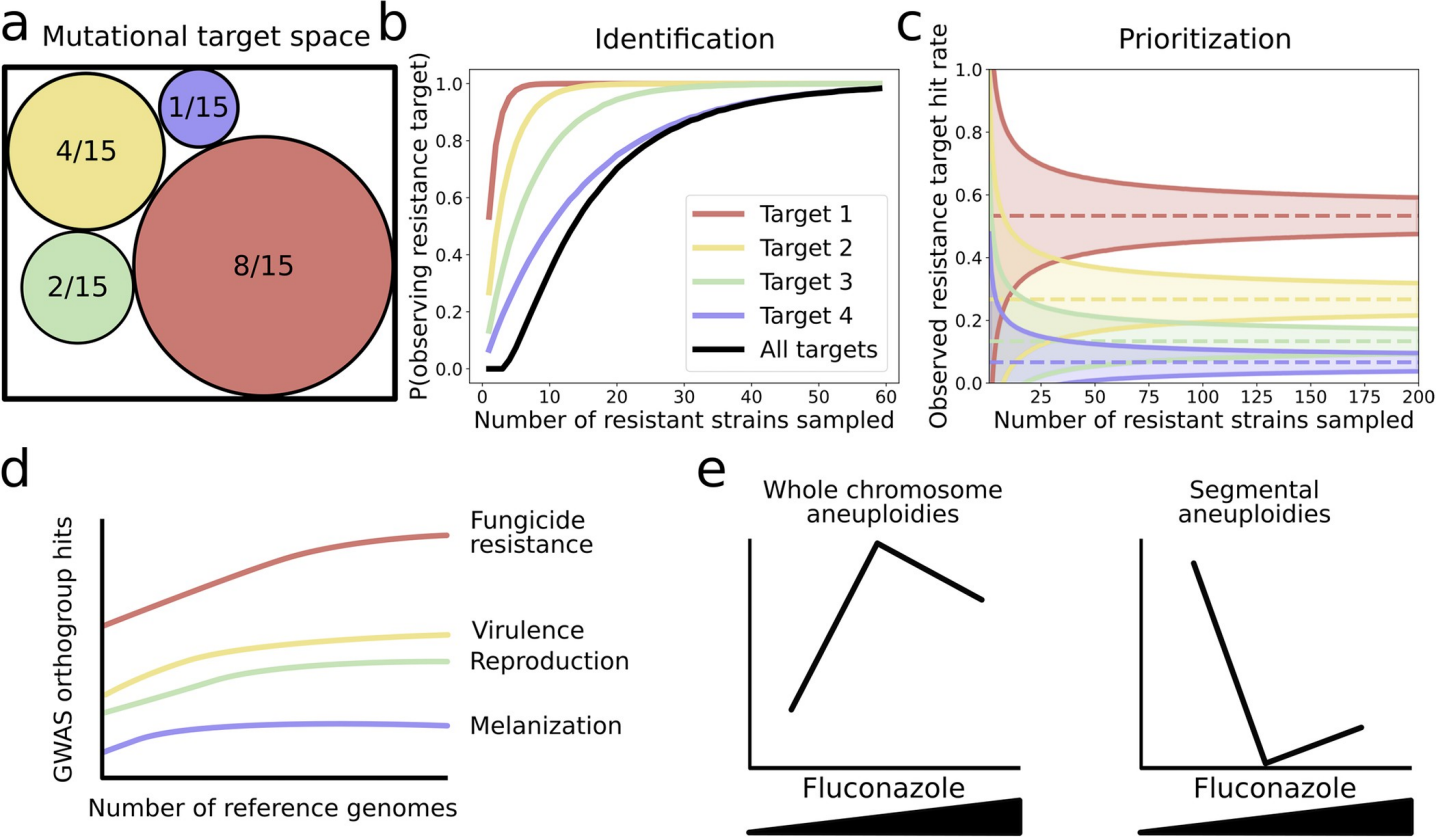
Increased sampling improves resistance target discovery and reveals evolutionary patterns. (a) Mock scenario showcasing the impact of sampling depth in genomics or experimental evolution studies. Here, mutations in 4 different genes can lead to resistance but occur at different rates for each target: ptarget 1 = 8/15, ptarget 2 = 4/15, ptarget 3 = 2/15, and ptarget 4 = 1/15. (**b**) The chance of detecting each target as a function of sampling as modeled by a multinomial distribution. While both high-frequency targets have approximately 100% chance of being detected in under 10 samples, having a more than 90% chance of identifying all genes requires>40 samples. (**c**) If trying to prioritize the most common driver of resistance for downstream studies or interventions, low sample size can lead to misleading conclusions. Estimating the relative contribution of each target is difficult if sampling is limited, as shown by the large overlap between the 5th to 95th percentiles of hit rates measured below 25 samples. (**d**) Additional reference genomes for SNP mapping increase the number of associations with resistance and virulence phenotypes. Dutta and colleagues [12] performed a GWAS for 49 life history traits on a panel of 145 *Zymoseptoria tritici* strains. Including additional reference genomes for SNP mapping increased the number of significant orthogroup-trait associations by up to a third. (**e**) Experimental evolution along an antifungal gradient uncovers dose-dependent effects on structural variation. Todd and colleagues [13] evolved *Candida albicans* strains at different fluconazole concentrations and found that while whole chromosome aneuploidies were more common at high concentrations in the SC5314 genetic background, this pattern was reversed for segmental aneuploidies, hinting at differences in fitness trade-offs for these 2 types of structural variants.

In the years since the first fungal genomes were published, the simultaneous reduction in cost and increase in throughput of next-generation sequencing has made genome sequencing far more accessible. Most fungal pathogens now have a reference genome, with telomere-to-telomere assemblies achievable from long-read sequencing. These high-quality references facilitate the identification of genetic variation associated with resistance phenotypes. In this context, large-scale sequencing of isolates is key to reliably identifying mutational resistance targets and understanding their relative importance (Fig 1A–1C). These analyses can also be combined with other phenotypic measurements to perform genome-wide association studies (GWAS) examining resistance, pathogenicity traits, or disease outcomes [10,11]. Such efforts can benefit from expanding reference genome data sets to include a higher level of diversity for variant mapping, as shown by a recent study on the wheat pathogen *Zymoseptoria tritici* (Fig 1D, [12]).

Large genome data sets can also help identify either shared or unique pathways to resistance among strains and species. At the species level, sets of high-quality reference genomes (>15) can help catalog variation in gene repertoire potentially linked to resistance or virulence [14,15], or allow machine-learning algorithms to build models to predict fungal lifestyles [16]. High-throughput sequencing of isolates is also helpful in mapping out epidemiological relationships and tracking drug resistance emergence during disease outbreaks in humans and plants [17–19]. Even within patient samples, genome sequencing can quantify genetic diversity during infection and help identify variations in resistance phenotypes. For example, sequencing multiple *Candida glabrata* (*Nakaseomyces glabratus*) isolates per blood culture sample revealed substantial genotypic and phenotypic diversity within blood infections, including fluconazole-resistant subpopulations [20]. Sequencing of environmental isolates can also allow for surveillance of alleles of concern which is especially relevant in scenarios where cross-resistance is suspected or has been shown. For example, the same cyp51A variants provide resistance to agricultural and medical azoles in Aspergillus fumigatus, leading to the acquisition of resistant infections from the environment [4]. Large sequencing projects thus remain important to understand the ecology and evolution of resistance and are often the first step in characterizing emerging pathogens of concern [21].

The study of resistance is not limited to strains isolated from clinical and environmental settings. High-throughput experiments where many populations are evolved in parallel are now routinely performed and can provide a better picture of the landscape of genes involved in resistance. For example, a recent study evolved almost 300 *C. glabrata* lineages, starting from different genetic backgrounds and under multiple antifungal exposure regimens, identifying multiple recurrently mutated genes [22]. When combined with accurate fitness measurements, large sets of independently generated resistant strains can be genotyped and phenotyped systematically to investigate fitness costs of resistance: in this case, many lineages evolved resistance with only mild reductions in growth rate. Natural experiments such as *in vivo* time course isolate sequencing can provide insights into adaptation to host conditions [23] or identify genetic events that correlate with the emergence of resistance [24], without the biases that might be linked with *in vitro* evolution conditions.

Additionally, using this approach, fundamental questions involving the evolvability of resistance can be tested quantitatively: for example, how different genetic backgrounds influence the emergence of resistance [25], or how the concentration of antifungal used in experimental evolution impacts which resistance alleles are observed [13,26]. For example, Todd and colleagues [13] found that fluconazole concentration influenced the rate of evolution of types of structural variants (Fig 1E). In another case, cross-feeding between cells in culture was found to dramatically alter the mutational targets of 5-Fluorocytosine (5-FC, flucytosine) resistance in yeast [27]. In the future, adapting pooled experimental evolution with lineage tracing to pathogenic fungi could allow for even higher throughput and more exhaustive mapping of different phenotype clusters associated with resistance or multidrug resistance [28]. By discovering new genes mediating resistance and providing quantitative information on the evolvability of phenotypes, large-scale experimental evolution provides unique knowledge of the different trajectories by which resistant strains can arise. These analyses can then provide larger sets of candidate genes for downstream functional characterization.

## Synthetic biology tools provide new avenues for reverse genetics in pathogens

In recent years, the explosion of genome-editing resources in fungi has allowed research groups to expand the functional genomics of antifungal resistance to new species where genetic manipulation was previously more cumbersome. In species like *Cryptococcus neoformans* and *Yarrowia lipolytica*, this has even led to the development of large-scale gene disruption assays [29,30] akin to those commonly performed in mammalian cells. CRISPR-Cas9 systems have been widely adopted to facilitate the study of human and plant pathogens. The targeted nature of CRISPR-Cas9 editing also led to the development of gene drive systems in *Candida albicans*, which can be used in conjunction with haploid strains for genetic interaction mapping [31]. This approach was then used to systematically explore the role of efflux pumps on antifungal resistance, finding several negative genetic interactions resulting in increased antifungal sensitivity. Another powerful approach to studying gene function in fungi uses transposon insertion mutagenesis coupled with high-throughput sequencing of insertion site flanking regions (Tn-seq) to detect genomic regions important to fitness [32]. This method has been adapted to map essential genes for pathogens of interest such as *A. fumigatus* [33] and *C. albicans* [34], where it was also used to identify genes linked with fluconazole resistance [35].

**Fig 2:**
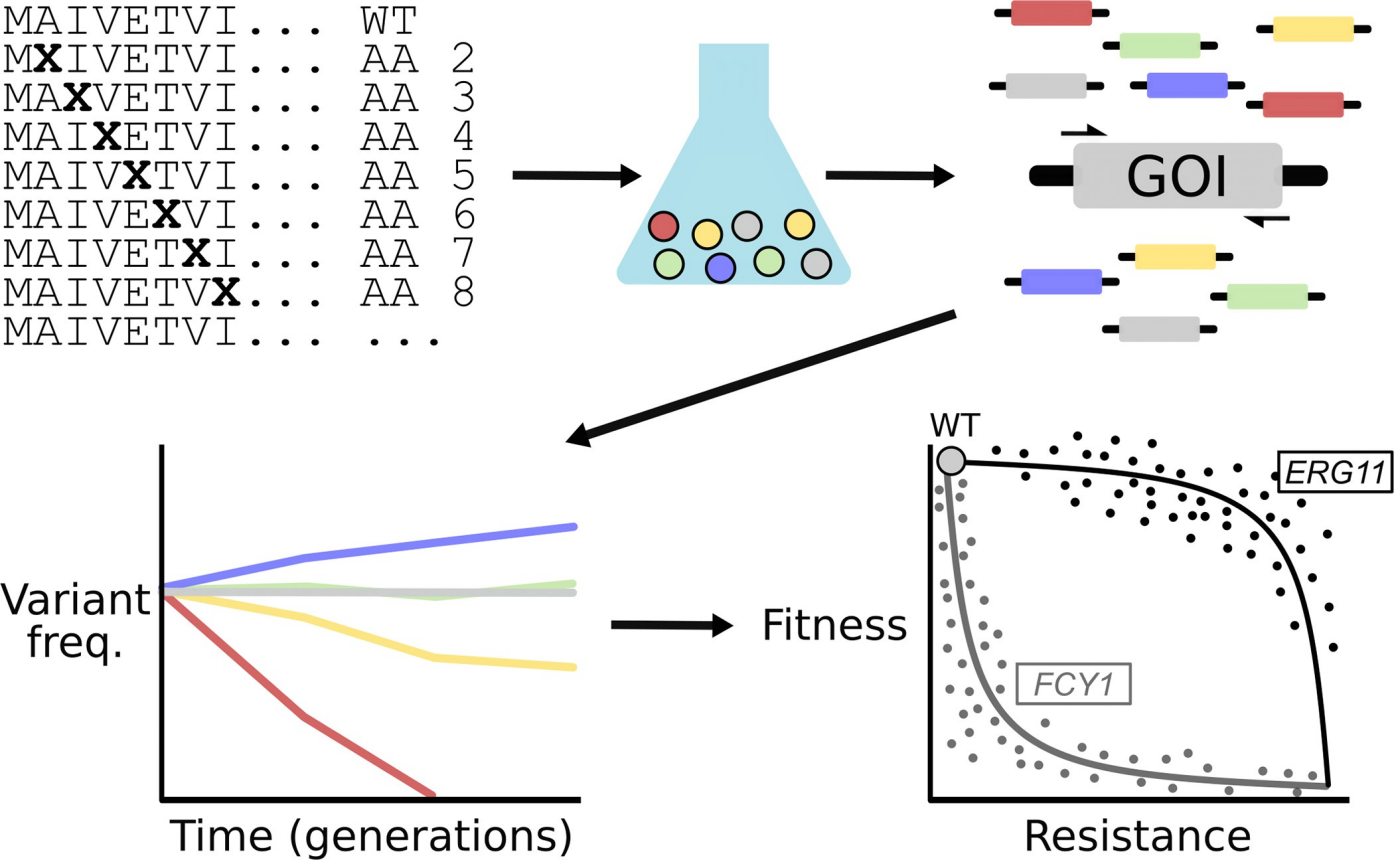
MAVEs systematically characterize the phenotypes of protein variants. The coding sequence of a gene of interest (GOI) is mutagenized to generate a library of mutant alleles, usually resulting in 1 amino acid substitution per protein. These alleles are then batch-transformed into a recipient strain to generate a large pool of variants that can be competed against one another under selective pressure like antifungal exposure. The abundance of each variant can be tracked by deep sequencing the locus of interest or a DNA barcode region that serves as an identifier for variants. By following the relative abundance in sequencing data of each variant at different pooled competition time points and comparing it to those of wild-type alleles, the fitness of each variant can be inferred. By modulating experimental conditions, the effect of variants on both resistance and fitness can be measured and the trade-off between the 2 can be characterized. In the case of the azole target *ERG11* [37], most resistant variants were found to have little impact on fitness, resulting in next to no compromise between the two. Conversely, mutations in the 5-FC target *FCY1* [36] granting even a small amount of resistance resulted in an almost full loss of function, signaling a strong resistance-fitness trade-off.

Advances in synthetic biology also allow researchers to leverage systems biology approaches to study antifungal resistance. Multiplexed assays of variant effects (MAVEs) are large-scale experiments that aim to test the effect of thousands of protein variants of a gene of interest at once (Fig 2). At the moment, 2 MAVE experiments have been performed on classical antifungal drug targets, both highlighting different approaches. The first used CRISPR-Cas9 to introduce mutant alleles of one of the causal genes implicated in 5-FC resistance at the endogenous yeast locus [36]. This allowed the authors to identify over 900 missense variants associated with resistance. In the second, a library of plasmids encoding mutant alleles of *C. albicans ERG11* expressed in a *Saccharomyces cerevisiae* strain where the endogenous copy was under the control of a repressible promoter was used to assay over 4,000 amino acid variants for resistance to 6 different azoles [37]. In both cases, resistance and function were measured in parallel for variants, allowing for the measurement of functional trade-offs. MAVE scores accurately predicted most phenotypes associated with orthologous mutant *FCY1* and *ERG11* alleles from fungal pathogens. While this has not been tested systematically yet, these assays provide a cost-effective avenue to catalog resistance alleles at high throughput.

In addition, heterologous MAVE assays can also allow for the study of resistance variants from species that have been impossible to cultivate in the lab, like *DFR1* from *Pneumocystis jirovecii* which could mediate sensitivity to methotrexate [38]. As more genes implicated in resistance and virulence are uncovered, MAVEs will provide insights into their function and help characterize trade-offs associated with resistance. These assays also facilitate the interpretation of genome sequencing data by generating sets of “variants of concern” for downstream applications like surveillance, thus contributing to the fight against resistance. Across almost all fungal pathogens, synthetic biology tools are poised to accelerate the pace of research and discovery by facilitating molecular genetics and enabling large-scale functional genomics in more species in the coming years.

## The future: Community resources to maximize data usefulness

Despite the progress made in identifying genomic features involved in antifungal resistance, much of this knowledge remains dispersed between databases dedicated to different organisms. One key resource that would facilitate access to current and future information is an extensive, trusted resistance mutation database for fungi, similar to what the Comprehensive Antibiotic Resistance Database (CARD) provides for prokaryotes [39]. The first iteration of a similar resource for fungi, the Mycology Antifungal Resistance Database (MARDy), contains approximately 230 entries covering literature up to 2018 [40]. A promising avenue to accelerate literature curation is the use of machine learning to automate partial or total information retrieval from research articles [41]. This approach was recently used to extract information on antifungal resistance, but the resulting annotations do not entirely overlap with the manual curation results from MARDy and have not always been de-duplicated [42]. In both cases, the annotations are mostly focused on human pathogens, despite the importance of resistance in the context of plant pathogens.

Beyond data on mutational effects, central repositories for gene function annotation and phenotypes are useful tools to facilitate meta-analysis, as is ongoing at FungiDB, the Candida Genome Database (CGD) and the Saccharomyces Genome Database (SGD) [43–45]. This will require efforts to compile and better integrate functional assays or gene expression data sets and the associated metadata. While this type of curation can be labor-intensive, computer-assisted methods may be able to accelerate this process [41]. Improving the way we store and use the large data sets that will come out of the next generation of functional genomics assay will give us the best chance of finding new approaches to tackle antifungal resistance. Guidelines for the interpretation of functional assay data by clinicians performing genetic testing in humans [46] could be adapted to the context of antifungal resistance to facilitate the use of this knowledge by infectious disease specialists. Independent of the type of assay, ensuring that detailed metadata is available for each annotation will be key to making reuse possible for a wide group of users. Our ability to study fungal biology at scale has accelerated rapidly in recent years, and the integration of other high-throughput methods like single-cell sequencing will further bolster our ability to dissect the mechanisms behind antifungal resistance. These new tools will undoubtedly accelerate the pace of antifungal resistance research and ultimately our ability to tackle these resistant infections as their frequency increases.

## References

[1] Denning DW. Global incidence and mortality of severe fungal disease. Lancet Infect Dis. 2024. doi: 10.1016/S1473-3099(23)00692-8 38224705

[2] One Health: Fungal Pathogens of Humans, Animals, and Plants. American Society for Microbiology; 2019. 31769941

[3] WHO fungal priority pathogens list to guide research, development and public health action. World Health Organization; 25 Oct 2022 [cited 2024 Mar 29]. Available from: https://www.who.int/publications/i/item/9789240060241.

[4] Rhodes J, Abdolrasouli A, Dunne K, Sewell TR, Zhang Y, Ballard E, et al. Population genomics confirms acquisition of drug-resistant Aspergillus fumigatus infection by humans from the environment. Nat Microbiol. 2022;7:663–674. doi: 10.1038/s41564-022-01091-2 35469019 PMC9064804

[5] Fisher MC, Alastruey-Izquierdo A, Berman J, Bicanic T, Bignell EM, Bowyer P, et al. Tackling the emerging threat of antifungal resistance to human health. Nat Rev Microbiol. 2022;20:557–571. doi: 10.1038/s41579-022-00720-1 35352028 PMC8962932

[6] Revie NM, Iyer KR, Robbins N, Cowen LE. Antifungal drug resistance: evolution, mechanisms and impact. Curr Opin Microbiol. 2018;45:70–76. doi: 10.1016/j.mib.2018.02.005 29547801 PMC6135714

[7] Kidd SE, Chen SC-A, Meyer W, Halliday CL. A New Age in Molecular Diagnostics for Invasive Fungal Disease: Are We Ready? Front Microbiol. 2019;10:2903. doi: 10.3389/fmicb.2019.02903 31993022 PMC6971168

[8] Hoenigl M, Sprute R, Egger M, Arastehfar A, Cornely OA, Krause R, et al. The Antifungal Pipeline: Fosmanogepix, Ibrexafungerp, Olorofim, Opelconazole, and Rezafungin. Drugs. 2021;81:1703–1729. doi: 10.1007/s40265-021-01611-0 34626339 PMC8501344

[9] Gabaldón T. Nothing makes sense in drug resistance except in the light of evolution. Curr Opin Microbiol. 2023;75:102350. doi: 10.1016/j.mib.2023.102350 37348192

[10] Sephton-Clark P, Tenor JL, Toffaletti DL, Meyers N, Giamberardino C, Molloy SF, et al. Genomic Variation across a Clinical Cryptococcus Population Linked to Disease Outcome. MBio. 2022;13:e0262622. doi: 10.1128/mbio.02626-22 36354332 PMC9765290

[11] Amezrou R, Ducasse A, Compain J, Lapalu N, Pitarch A, Dupont L, et al. Quantitative pathogenicity and host adaptation in a fungal plant pathogen revealed by whole-genome sequencing. Nat Commun. 2024;15:1933. doi: 10.1038/s41467-024-46191-1 38431601 PMC10908820

[12] Dutta A, McDonald BA, Croll D. Combined reference-free and multi-reference based GWAS uncover cryptic variation underlying rapid adaptation in a fungal plant pathogen. PLoS Pathog. 2023;19:e1011801. doi: 10.1371/journal.ppat.1011801 37972199 PMC10688896

[13] Todd RT, Soisangwan N, Peters S, Kemp B, Crooks T, Gerstein A, et al. Antifungal Drug Concentration Impacts the Spectrum of Adaptive Mutations in Candida albicans. Mol Biol Evol. 2023:40. doi: 10.1093/molbev/msad009 36649220 PMC9887641

[14] McCarthy CGP, Fitzpatrick DA. Pan-genome analyses of model fungal species. Microb Genom. 2019:5. doi: 10.1099/mgen.0.000243 30714895 PMC6421352

[15] Badet T, Oggenfuss U, Abraham L, McDonald BA, Croll D. A 19-isolate reference-quality global pangenome for the fungal wheat pathogen Zymoseptoria tritici. BMC Biol. 2020;18:12. doi: 10.1186/s12915-020-0744-3 32046716 PMC7014611

[16] Dort EN, Layne E, Feau N, Butyaev A, Henrissat B, Martin FM, et al. Large-scale genomic analyses with machine learning uncover predictive patterns associated with fungal phytopathogenic lifestyles and traits. Sci Rep. 2023;13:17203. doi: 10.1038/s41598-023-44005-w 37821494 PMC10567782

[17] Rhodes J, Abdolrasouli A, Farrer RA, Cuomo CA, Aanensen DM, Armstrong-James D, et al. Genomic epidemiology of the UK outbreak of the emerging human fungal pathogen Candida auris. Emerg Microbes Infect. 2018;7:43. doi: 10.1038/s41426-018-0045-x 29593275 PMC5874254

[18] Chow NA, Muñoz JF, Gade L, Berkow EL, Li X, Welsh RM, et al. Tracing the Evolutionary History and Global Expansion of Candida auris Using Population Genomic Analyses. MBio. 2020:11. doi: 10.1128/mBio.03364-19 32345637 PMC7188998

[19] Latorre SM, Were VM, Foster AJ, Langner T, Malmgren A, Harant A, et al. Genomic surveillance uncovers a pandemic clonal lineage of the wheat blast fungus. PLoS Biol. 2023;21:e3002052. doi: 10.1371/journal.pbio.3002052 37040332 PMC10089362

[20] Badrane H, Cheng S, Dupont CL, Hao B, Driscoll E, Morder K, et al. Genotypic diversity and unrecognized antifungal resistance among populations of Candida glabrata from positive blood cultures. Nat Commun. 2023;14:5918. doi: 10.1038/s41467-023-41509-x 37739935 PMC10516878

[21] Huang J, Hu P, Ye L, Shen Z, Chen X, Liu F, et al. Pan-drug resistance and hypervirulence in a human fungal pathogen are enabled by mutagenesis induced by mammalian body temperature. Nat Microbiol. 2024;9:1686–1699. doi: 10.1038/s41564-024-01720-y 38898217

[22] Ksiezopolska E, Schikora-Tamarit MÀ, Beyer R, Nunez-Rodriguez JC, Schüller C, Gabaldón T. Narrow mutational signatures drive acquisition of multidrug resistance in the fungal pathogen Candida glabrata. Curr Biol. 2021;31:5314–5326.e10. doi: 10.1016/j.cub.2021.09.084 34699784 PMC8660101

[23] Sephton-Clark P, McConnell SA, Grossman N, Baker RP, Dragotakes Q, Fan Y, et al. Similar evolutionary trajectories in an environmental Cryptococcus neoformans isolate after human and murine infection. Proc Natl Acad Sci U S A. 2023;120:e2217111120. doi: 10.1073/pnas.2217111120 36603033 PMC9926274

[24] Ford CB, Funt JM, Abbey D, Issi L, Guiducci C, Martinez DA, et al. The evolution of drug resistance in clinical isolates of Candida albicans. Elife. 2015;4:e00662. doi: 10.7554/eLife.00662 25646566 PMC4383195

[25] Gerstein AC, Berman J. Candida albicans Genetic Background Influences Mean and Heterogeneity of Drug Responses and Genome Stability during Evolution in Fluconazole. mSphere. 2020:5. doi: 10.1128/mSphere.00480-20 32581072 PMC7316494

[26] Yang F, Scopel EFC, Li H, Sun L-L, Kawar N, Cao Y-B, et al. Antifungal Tolerance and Resistance Emerge at Distinct Drug Concentrations and Rely upon Different Aneuploid Chromosomes. MBio. 2023;14:e0022723. doi: 10.1128/mbio.00227-23 36877011 PMC10127634

[27] Durand R, Jalbert-Ross J, Fijarczyk A, Dubé AK, Landry CR. Cross-feeding affects the target of resistance evolution to an antifungal drug. PLoS Genet. 2023;19:e1011002. doi: 10.1371/journal.pgen.1011002 37856537 PMC10617708

[28] Schmidlin K, Apodaca S, Newell D, Sastokas A, Kinsler G, Geiler-Samerotte K. Distinguishing mutants that resist drugs via different mechanisms by examining fitness tradeoffs across hundreds of fluconazole-resistant yeast strains. Elife. 2024. doi: 10.7554/elife.94144.1 PMC11386965 39255191

[29] Li Z, Kim KS. RELATe enables genome-scale engineering in fungal genomics. Sci Adv. 2020:6. doi: 10.1126/sciadv.abb8783 32948588 PMC7500931

[30] Schwartz C, Cheng J-F, Evans R, Schwartz CA, Wagner JM, Anglin S, et al. Validating genome-wide CRISPR-Cas9 function improves screening in the oleaginous yeast Yarrowia lipolytica. Metab Eng. 2019;55:102–110. doi: 10.1016/j.ymben.2019.06.007 31216436

[31] Shapiro RS, Chavez A, Porter CBM, Hamblin M, Kaas CS, DiCarlo JE, et al. A CRISPR–Cas9-based gene drive platform for genetic interaction analysis in Candida albicans. Nat Microbiol. 2017;3:73–82. doi: 10.1038/s41564-017-0043-0 29062088 PMC5832965

[32] van Opijnen T, Bodi KL, Camilli A. Tn-seq: high-throughput parallel sequencing for fitness and genetic interaction studies in microorganisms. Nat Methods. 2009;6:767–772. doi: 10.1038/nmeth.1377 19767758 PMC2957483

[33] Carr PD, Tuckwell D, Hey PM, Simon L, d’Enfert C, Birch M, et al. The transposon impala is activated by low temperatures: use of a controlled transposition system to identify genes critical for viability of Aspergillus fumigatus. Eukaryot Cell. 2010;9:438–448. doi: 10.1128/EC.00324-09 20097738 PMC2837977

[34] Segal ES, Gritsenko V, Levitan A, Yadav B, Dror N, Steenwyk JL, et al. Gene Essentiality Analyzed by In Vivo Transposon Mutagenesis and Machine Learning in a Stable Haploid Isolate of Candida albicans. MBio. 2018:9. doi: 10.1128/mBio.02048-18 30377286 PMC6212825

[35] Gao J, Wang H, Li Z, Wong AH-H, Wang Y-Z, Guo Y, et al. Candida albicans gains azole resistance by altering sphingolipid composition. Nat Commun. 2018;9:4495. doi: 10.1038/s41467-018-06944-1 30374049 PMC6206040

[36] Després PC, Cisneros AF, Alexander EMM, Sonigara R, Gagné-Thivierge C, Dubé AK, et al. Asymmetrical dose responses shape the evolutionary trade-off between antifungal resistance and nutrient use. Nat Ecol Evol. 2022;6:1501–1515. doi: 10.1038/s41559-022-01846-4 36050399

[37] Bédard C, Gagnon-Arsenault I, Boisvert J, Plante S, Dubé AK, Pageau A, et al. Most azole antifungal resistance mutations in the drug target provide cross-resistance and carry no intrinsic fitness cost. bioRxiv. 2023. p. 2023.12.13.571438. doi: 10.1101/2023.12.13.571438

[38] Rouleau FD, Dubé AK, Gagnon-Arsenault I, Dibyachintan S, Pageau A, Després PC, et al. Deep mutational scanning of Pneumocystis jirovecii dihydrofolate reductase reveals allosteric mechanism of resistance to an antifolate. bioRxiv. 2023. p. 2023.09.27.559786. doi: 10.1101/2023.09.27.559786 PMC11125491 38683847

[39] Alcock BP, Huynh W, Chalil R, Smith KW, Raphenya AR, Wlodarski MA, et al. CARD 2023: expanded curation, support for machine learning, and resistome prediction at the Comprehensive Antibiotic Resistance Database. Nucleic Acids Res. 2023;51:D690–D699. 36263822 10.1093/nar/gkac920 PMC9825576

[40] Nash A, Sewell T, Farrer RA, Abdolrasouli A, Shelton JMG, Fisher MC, et al. MARDy: Mycology Antifungal Resistance Database. Bioinformatics. 2018;34:3233–3234. doi: 10.1093/bioinformatics/bty321 29897419 PMC6137992

[41] Edalatmand A, McArthur AG. CARD*Shark: automated prioritization of literature curation for the Comprehensive Antibiotic Resistance Database. Database. 2023;2023. doi: 10.1093/database/baad023 37079891 PMC10118295

[42] Jain A, Singhal N, Kumar M. AFRbase: a database of protein mutations responsible for antifungal resistance. Bioinformatics. 2023:39. doi: 10.1093/bioinformatics/btad677 37947313 PMC10656092

[43] Skrzypek MS, Binkley J, Binkley G, Miyasato SR, Simison M, Sherlock G. The Candida Genome Database (CGD): incorporation of Assembly 22, systematic identifiers and visualization of high throughput sequencing data. Nucleic Acids Res. 2017;45:D592–D596. doi: 10.1093/nar/gkw924 27738138 PMC5210628

[44] Alvarez-Jarreta J, Amos B, Aurrecoechea C, Bah S, Barba M, Barreto A, et al. VEuPathDB: the eukaryotic pathogen, vector and host bioinformatics resource center in 2023. Nucleic Acids Res. 2024;52:D808–D816. doi: 10.1093/nar/gkad1003 37953350 PMC10767879

[45] Wong ED, Miyasato SR, Aleksander S, Karra K, Nash RS, Skrzypek MS, et al. Saccharomyces genome database update: server architecture, pan-genome nomenclature, and external resources. Genetics. 2023:224. doi: 10.1093/genetics/iyac191 36607068 PMC10158836

[46] Gelman H, Dines JN, Berg J, Berger AH, Brnich S, Hisama FM, et al. Recommendations for the collection and use of multiplexed functional data for clinical variant interpretation. Genome Med. 2019;11:85. doi: 10.1186/s13073-019-0698-7 31862013 PMC6925490

